# Oncogenic potential of Human Papillomavirus (HPV) and its relation with cervical cancer

**DOI:** 10.1186/1743-422X-8-269

**Published:** 2011-06-03

**Authors:** Rabia Faridi, Amreen Zahra, Khalida Khan, Muhammad Idrees

**Affiliations:** 1 Division of Molecular Virology, National Centre of Excellence in Molecular Biology, University of the Punjab, 87-West Canal Bank Road Lahore-53700, Pakistan; 2 Center for Integrated Mountain Research, University of the Punjab New Campus Lahore-54590, Pakistan

## Abstract

Human Papillomavirus (HPV) is the most common cause of cervical cancer. Cervical cancer being the second most common cancer after lung cancer, affecting women of different age groups; has a prevalence of about 20% in young sexually active women. Among different types of HPV, HPV16 the major strain causing this cancer and is sexually transmitted had been unnoticed for decades. Keeping in mind the multiple risk factors related with cervical cancer such as early age sexual activities, teenage pregnancies, smoking, use of oral contraceptives, having multiple sex partners, hormone replacement therapies and various other unknown factors lead to the onset of the disease. Awareness for various diagnostic procedures such as Pap smears screening prove to be an effective way in eradicating the oncogenic potential of HPV.

## Background

Human papilloma viruses (HPV) are small non enveloped virus containing double stranded DNA as their genetic material and are about 55 nm in size. Their genome has three functional coding regions; E: a gene coding early viral function, L: a gene coding late viral function and LCR: a long control region which lies between E and L. They appear to show similarity with plyoma viruses and consist of about 72 capsomeres. The papilloma viruses and its viral nature were first seen in human warts in 1907, and the first papilloma virus was isolated from a rabbit that was identified by Richard Shoppe in the year 1983. Even it was an early start for the detection of human papilloma viruses; this topic remained closed till 1970's. Studies related with papilloma viruses were allowed to move forth only when cervical cancer's proximity with HPV was proved and with its increasing significance in the field of molecular virology [1].

Human papilloma virus is known to be the main reason for cervical carcinomas. Back in 1976 HPV was seen with its involvement in the successful development of cervical tumours and since then various epidemiological and biological statistics has justified this observation [2]. HPV viruses are one of the most common viruses which are transmitted sexually and are found in both men and women. Its ratio is much higher in western countries as compared to other regions of the world.

### Prevalence and Incidence

Cervical cancer as the most prevalent cancer in women in the coming years is the main cause of death, especially in young women. Its ratio varies all over the world, but a significantly high ratio has been seen in the western countries. By statistical and geographical analysis it proves to be higher in Native American women than in Caucasian women. It was also found common in Hispanic and African American women [3]. According to the World Health Organization (WHO) statistics, there are approximately 500,000 new cases registered each year out of which 250,000 cases are fatal. This alarming situation in the coming years for papillomaviruses has lead molecular virologists world wide to go deep into pathogenesis and bring out solutions to its therapeutic potential [4]. According to the most recent reports, in the United States of America (USA) women are prone to this infection twice than men in all regions of the world including USA and the prevalence of HPV is much higher in women than men. The reported overall prevalence rate of HPV in women irrespective of races was 17.9%, while men showed a comparably lower rate of 8%. African Americans had the ratio of 20 to 29% as Caucasians and their prevalence rate was known to be about 12.5%. People who had multiple sex partners during their lifetime were on a higher risk of HPV infection as the frequency of HPV was seen to be 20.1% compared 7% in those patients who had only one sexual partner throughout their sexual lives [5].

### Different types of HPV

About 100 different subtypes of HPV with distinguished variations in its genetic and oncogenic potential are known. The subtypes which specifically affect the anogenital tract are HPV subtypes 16, 18, 31, 33, 35, 39, 45, 51, 52, 56, 58, 66 and 69 [3]. These specific subtypes can be further classified or described under high-risk and low-risk parameters. These specific subtypes possess a remarkable ability to cause malignancy and play a pivotal role in development of various tumours [2]. The HPV type 6 and 11 are considered as the low risk genital types while on the other hand HPV 16 and 18 contribute towards malignancy and fall under the high risk genital types [6]. Prior to a confirmed malignancy of the cervical region, there appears a cervical intraepithelial neoplasia (CIN) which can indicate it towards a high risk HPV infection and this infection is actually the prolonged interval with specific symptoms till the development of a tumour [2]. It is now an established fact that papillomaviruses are highly species specific.

### Structure of HPV 16

**Figure 1:**
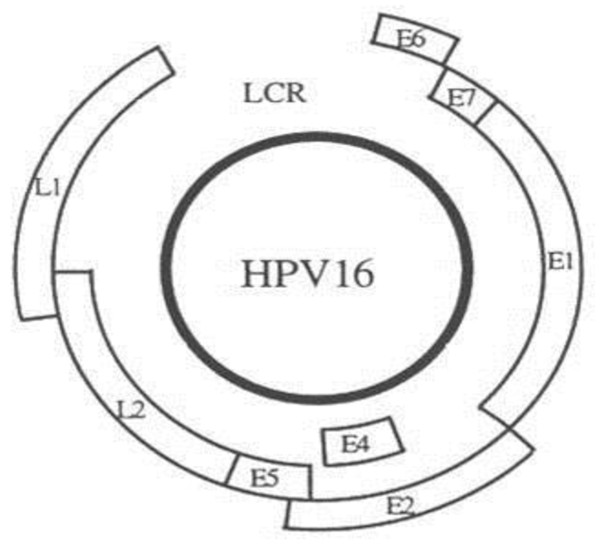
**The figure shows the location of the major open reading frames which possess the ability to encode proteins and the long control regions which have the ability of transcription and replication control elements (Adapted from Molecular biologists for Oncologists.1996, 2^nd ^edd., Editors "J.R.Yarnold, M.R.Stratton, T.J.McmMillan" publishers, Chapman and Hall)**.

HPV16 is one of the most prominent strains of HPV which is typically classified under high risk type infection as mentioned earlier. It is associated with additional infections namely lichen sclerosis and not only it leads to cervical cancer but is also related with vaginal, vulval, anal and penile cancers or benign growths specifically in these areas, but the most prominent and hazardous damage caused by HPV 16 strain is cervical cancer [7]. The HPV 16 is a small non enveloped virus containing double stranded DNA as its genetic material. Its genome has seven functional coding regions; E1/E2: Code proteins that control the function of E6 and E7 genes, E4: a gene coding a protein the function of this protein is largely unknown but may control virus release from cell, E5: codes for a hydrophobic protein which enhances immortalization of the cell, E6: gene coding proteins which inhibit negative regulators of the cell cycle and further inhibit p53 which is a transcription factor for apoptosis, E7: codes for viral protein that bind to the retinoblastoma tumor suppressor proteins thereby permitting the cell to progress through the cell cycle in the absence of normal mitogenic signals, L1/L2: Code for structural proteins for late viral function and formation of complete virus particles and LCR: a long control region which lies between E and L that is necessary for normal virus replication and control of gene expression (Figure 1).

### Pathogenesis of HPV 16 and its Replication

Replication of HPV 16 is initiated with the replication of viral DNA in contrast with the cellular chromosomes. In this process of division, one daughter cell moves apart from the basal lamina to undergo differentiation. Infection in the basal lamina can persist for years because, of the restriction of virion production to only differentiated cells. The E_1 _and E_2 _proteins are supposed to be the recognition factors and regulators for early viral transcription. Icosahedral capsid for virion generation are formed by L_1 _and L_2 _[1].

HPV 16 affects approximately 20% of the adult population particularly in western countries [8]. It sometimes causes warts which are flat in appearance called condyloma planum and they appear to be less visible. Others include cauliflower like warts mostly seen in the genital areas [9]. The three major onco-proteins which have gained importance in HPV and its relation with cervical cancer are E_5_, E_6_, E_7 _respectively. These proteins are associated with cervical cancer studied both *in vitro *and *in vivo *[10]. The proteins E_6 _and E_7 _are important for the inhibition of tumour suppressor genes namely p53 and pRb [11]. As far as E_5 _is concerned it is known to have weak oncogenic properties which result in increased activity of epidermal growth factor receptor (EGFR) and there by inhibiting the major histocompatibility complex expression [11]. For a crucial step to initiate in carcinogenesis it is very necessary that the viral DNA integrates into the host genome. To understand this concept of integration leading to malignancy it is very important to jot down that E_2 _and E_1 _are the two proteins which play a leading role in control of transcription and replication and in the absence or loss of activity of these proteins deregulation of E_6 _and E_7 _onco-proteins results [12].

HPV I6 E_6 _protein comprises of about 151 amino acids, these proteins are confined to the nuclear matrix and non-nuclear membrane fraction. Their major function is in zinc binding. This E_6 _protein is related with high risk HPV associated with wild type p53 tumour suppressor protein. To get associated with p53, the E_6 _protein needs a cellular protein of about 100-kDa and is known as E_6_-AP [13]. The E_6 _protein favours abnormal cell growth by the process of binding and rapidly targeting the p53 protein [14]. This p53 protein shows degradation by the ubiquitin pathway and the cell is allowed to go from the p53 associated negative growth control [15]. The E_6_-E_6 _AP complex specifically target ubiquitin targeted substrates. It also shows p53 disruption and p53 mediated apoptosis [16]. HPV E_7 _proteins are small proteins consisting of 98 amino acids and zinc binding phosphoproteins are confined in the nucleus. This high risk HPV E_7 _protein has a higher binding affinity for pRb protein [17].

The mutational analysis reveals that an E7 protein plays a major role in transformation and has proved its ability with interference with pRb function. This interference of E7 protein with the pRb function contributes in the development of malignancy. pRb is also a tumour suppressor gene and is the main mutant allele of Rb present in the germline of an individual which is linked to a predisposition to retinal tumour [18]. The major finding that how pRb functions in growth control lies with the proof of its binding ability and inhibition of various transcription factors namely E_2 _F-1and cMyc proteins. These cMyc and E_2 _F-1 proteins activate some of the essential genes which are required for DNA synthesis. The HPV infected cell typically shows that pRb is inactive throughout in association with binding of the E_7 _proteins. This decreased or inappropriate release of transcription factors favours the deregulated expression of genes which are related with normal control and cell division [19].

More striking evidence reported by several studies is that E_7 _possesses and has the capability of overcoming DNA damage induced p53-dependant G_1 _arrest. In the precancerous lesions the loss of control point favours and allows the DNA damaged cells to enter the normal cell division initiating to cancerous or tumourigenic mutations. This evidence of mutations is well supported by recent reports that deregulate expression of E_2 _F in rat fibroblasts which leads to S phase entry in the cell cycle and apoptosis in p53 [20]. Moreover expression of high risk HPV oncogenes in this DNA damage response is most probably interfered or disrupted at two points. At first the E_6 _protein by targeting the p53 protein for degradation inhibits the transcriptional activation of the kinase inhibitor and secondly expression of the E_7 _proteins can result in the constitutive inactivation of pRb and hence the deregulation of E_2 _F_1 _takes place [20].

Many reviews and studies had focused on the mechanisms and significant properties of E_6 _and E_7 _proteins and it is very essential to bring into light the detailed analysis of these studies on the specific regions of HPV16 E_7 _proteins [6]. For mapping the contribution of these particular events of the protein sequence point mutations and deletions were used. In vitro studies have revealed that the biological properties of HPV16 E_7 _are also related with *in vivo *functions which include immortalization and transformation alone or in cooperation with *ras*.

### Causes of HPV16

As far as the causes of HPV16/HPV18 are concerned sexual behaviour is the strongest factor for this increasing malignancy. Despite sexual behaviour other factors might also be involved in its infection. It is important to note that all HPV infections does not lead to a malignancy however, the two strains of HPV that are HPV16 and HPV18 mainly contributes in the development of cervical cancer. Other factors include smoking, extensive use of oral contraceptives and history of having sexually transmitted diseases [21]. Mutations in *ras*, *fos *and other related oncogenes has also been detected in cervical cancer cell lines but their *in vivo *role and proliferation is still unpredicted [21].

Other factors that enhance cervical cancer include impaired immune response, persistence of virus, smoking and administration of steroid hormones [21]. Loss of tumour suppressor genes and the activation of oncogenes may also play an important role. For cervical carcinogenesis, steroid hormones (estrogens and progesterone) play a role in the initiation and successful development of progression of the disease. Women who take estradiol have been reported to develop HSIL lesions that ultimately lead to the progressive one [22,23]. Estrogen affects the immune response as well [24,25].

Haptoglobulin which was an acute phase glycoprotein present in body fluids is reported to be in relation with tumour and infection which modulate the Th1/Th2 response [26-29]. The same factors are considered responsible for the immune tolerance related to human fertility [26,27,30]. Apart from these mechanisms the genetic Hp polymorphism greatly enhances the female susceptibility for HPV cervical infection and neoplasia but also shows its relative response to the environmental cofactors such as sex steroid hormones ingestion which can be in the form of both hormone replacement therapy and oral contraceptive [26,27]. Keeping in view the other related factors the most prominent evidence for cervical cancer reported 80% of the studies are sexual behaviour and the changes related in sexual behaviour. The factor of sexual behaviour suffers lack of data, but according to the reported data on the subject, the increased ratio of CIN incidence among women ranging between 25 to 39 years. It may be due to the drastic changes in immigration, mixing patterns of population, population migration and economic migration [28].

### HPV 16 among men

HPV affects not only women, but this virus is even contracted by men. Men do have the ability to transmit it but unlike women in men there is no such confirmed lab test available such as women do have Pap smear tests [31].

### HPV16 among women

HPV16 which ultimately leads to cervical cancer stays and incubates in the body for a prolonged period of time before the onset of malignant cervical cancer [32]. Sometimes it stays inside the body for years. The reason of development of cervical cancer is that keratinocytes infected with HPV16 do not have the capability to differentiate like normal cells and they have close resemblance of just the precancerous cervical lesions, supporting the association between infection with specific high risk HPV types and the precancerous lesions. These cells are supposed to be not always malignant or with a tumorigenic potential but its induction to lead towards malignancy by the activation of certain oncogenes it can lead to malignancy [33]. They are now known to be the most prevalent etiological agents of cervical and various other anogenital malignancies. The human papilloma viruses are specifically released by the desquamating superficial keratinocytes of the infected stratified epithelia [34]. Its transmission can vary in aspects such as direct contact and indirect contact. Direct contact could be a result of contact with infected tissue and the indirect contact can be with contact of contaminated objects or surfaces for eg: pool sides. The transmission of genital HPV is related specifically with sexual contact. The viral entry leads to a latent infection which develops within the first three months or it can remain undetected for years. The lesions are due to spread because of the infected cells [35].

### Cervical cancer the 2^nd ^most malignancy and its relation with HPV16

Cervical cancer can also be defined as the cancer originating at the opening of the womb which progressively migrates to the whole of cervix [36]. HPV16 is known to cause cervical cancer and accounts for the majority of cervical carcinomas in United States and Europe [36,37]. HPV16 is roughly in half of the cervical cancers and their proteins have been detected extensively in HPV16 and are under observation. HPV 16 is one of the high risk HPV infections particularly and causes cervical lesions specifically. The two proteins which play a significant role in onset of this malignancy are E_6 _and E_7 _proteins and their expression has been seen in the cell lines [37]. IgG and IgA antibodies are associated with HPV16 and are found in CIN in a ratio of about 50-75%. Very few antibodies are detected in patients who have precancerous cervical lesion.

Back in 1977 statistical data suggested that HPV viruses are present in cervical epithelium [38]. In different cervical smears and biopsies from women who had CIN, koilocytes were noted which proved that wart virus antigen and HPV DNA nuclei of cervical epithelial cells are associated with cervical cancer. These observations lead to a platform for further research in this area [39]. The precancerous lesions of CIN2 and CIN3 have shown positive results for HPV high risk viruses that develop into invasive cancer. Apart from HPV 16 and HPV18, other strains such as HPV 31, 33, 35, 39, 45, 51, 52, 56, 58, 59 and 68 leads progressively to invasive cervical cancer whereas mostly low risk HPV types can lead to condylomata. Analysis from several recent reports state that HPV 16/18 have a remarkable ability to immortalize keratinocytes and exhibit a drastic pattern of differentiation.

The most striking and promising evidence that human papillomaviruses play a pivotal role in oncogenesis is based on different patterns of HPV DNA in cervical cells and its integration. The result of integration is disruption of E_1 _and E_2 _gene expression which control the genes and deregulation of E_6 _and E_7 _open reading frames. The normal function of tumour suppressor genes p53 and Rb is blocked by the products of E_6 _and E_7 _coding regions [40]. As a result of this blocking unchecked cellular proliferation takes place allowing the cell cycle check points to bypass through at G_1 _and G_2 _points. By this data it was confirmed that HPV16 provided the solid evidence for its development in cervical invasive cancer. HPV DNA was found and present in about 90% of all cervical cancers [41].

### Risk Factors

Keeping in view the association between HPV and Cervical Cancer, early age is thought to be a primary factor in HPV infection. Its probability increases with the number of sexual partners at any time of that person's life. Epidemiological data shows that the exogenous and endogenous sex hormones also make a women more prone in developing cervical cancer in combination with HPV infection [42]. By analysing different data on Cervical Cancer, It is indicated by having a check on number of sexual partners and age at Full Term Pregnancy (FTP). Cervical Cancer is more common in early pregnancies [43] and multiparity displayed changes of female hormones during pregnancy which showed high risk of Cervical Cancer in women having high parity. On the other hand Immune Suppression linked with pregnancy had more affinity for the risk of new HPV infections as in pregnancy; body is unable to eliminate the already existing infections making it more vulnerable to the risk of neoplastic progression.

Risk factor for Cervical Cancer also increases with the increase use of Oral Contraceptives (medications taken by mouth for the purpose of birth control). Women who use oral contraceptives more than 5 years of duration had a 3 fold increased in the development of cervical cancer than those who do not use such type of medications for birth control. To cap it all, according to epidemiological studies, the incidence of cervical cancer and increased risk in multifarious women and with women using hormonal contraceptives for a long time were consistent and confirm the hypothesis that female hormones favours the risk of cervical cancer. It was more common in women who avoided Pap smears and also in women who had orthodox religious beliefs such as church goers [44,45].

Human semen is also considered as the major biological basis in sexual behaviour that enhances HPV infection and cervical cancer. Chronic inflammation of cervix is due to local cellular and molecular changes that are reported to be triggered by multiple exposures to semen [46,47]. Human semen also interacts with cervical epithelial lining and cells which ultimately affects the synthesis of cytokines/chemokines which are involved in the immune response [48]. The most prominent prostaglandin PGE2 which is detected in semen play the role of a common inflammatory mediator. These prostaglandins are detected in 10,000 fold higher concentration at the site of inflammation [46,47,49]. Up-regulated PGE2 is considered to be the most favourable possible promoter of cervical carcinogenesis. Several studies showed that smoker's semen are concentrated with tobacco related carcinogens and play a role in causing cervical carcinogenesis [50,51]. Birth control procedures such as Tubal ligation and Intrauterine devices proved to be one of the most convincing methods in causing 80% of cervical cancer particularly in developing countries [52,53].

### Diagnosis

HPV infection can be clinically diagnosed by the presence of warts in low risk types. Genital warts which are sexually transmitted can be present in the form of cauliflower like smooth popular, small elevated warts. Progressive cervical disease initiates as a benign non invasive squamous intraepithelial lesion or CIM. Differential diagnosis can be done with three procedures of molecular assays namely: signal amplification, target amplification, non amplified hybridization assays [54]. Pap smears are the most commonly used lad diagnostic procedure for women. Cytological changes during HPV infection which are strongly associated with papaniclav (pap) smears screening, cell sampling of cervix is done and checked for signs of malignancy [54]. In pap diagnosis atypical squamous cells are of undetermined significance (ASCUS) during cervical screening. Although this diagnosis shows cell abnormality and is not sufficient for definitive diagnosis of a squamous intraepithelial lesion. Colposcpic biopsy is referred to women with these types of abnormalities [55].

### Vaccination

It is now well established fact that Cervical Cancer is due to HPV infection which is a sexually transmitted disease. Cervical cancer is among the second most common cancer in women worldwide. It is a multi-factorial disease. Various factors are involved in its progression of cervical cancers such as smoking, sexual behaviour, age and number of sexual partners [56-64]. Infection is highly linked to sexual activity with different partners. HPV associated genital infection are the most prevalent sexually transmitted infections in various countries including USA [65,66].

Risk of infection with HPV is directly proportional to age. Prophylactic HPV Vaccines is found to be effective in treating HPV infection and it is shown by number of randomized clinical trials [67-69]. Gardasil, a prophylactic vaccine, shows maximum efficacy if used by girls' pre-exposure to HPV. Prophylactic HPV Vaccines associated with VLPs and are made up of HPV L_1 _proteins at present. As VLPs, has no viral DNA therefore these are not associated with the development of infection even though their structure is similar to actual virus morphologically. By intra-muscular (I/M) injection, high levels of systemic anti HPV L_1_, Immunoglobulin G antibodies are formed due to VLPs [70].

Prophylactic HPV Vaccines targeting L_1 _viral protein which shows increased effectiveness and mostly adopted. Sexual naïve adolescents should be routinely vaccinated as recommended by World Health Organization (WHO). These vaccines can prove to be effective for future infection instead of previously exposed individuals. As Papillomavirus is non-lytic virus and can divide only in epithelial keratinocytes so, prophylactic vaccines seem to be ineffective in HPV neoplasia. Type of HPV is correlated with the clinical outcome of infection. For some infections, host's immune system is strong enough to eradicate and they get cleared instantly.

Several vaccination trials have shown that both bivalent against HPV 16, 18 and Quadrivalent against HPV 16, 18, 6 and 11 shows remarkable immunological response which is related to nearly 100% efficacy for a maximum 5 years in Naïve women [71-75]. Herd immunity is important in showing vaccine effects in women who are not vaccinated and in those who are reluctant to have cervical screening and receive vaccination at very young age. Due to this behaviour, non responders are about 50% of the screening population in Europe [76-80] and they are comprised of more than 50% cervical cancerous cases [81-85]. So, these non responders should be focused who are unable to get medical services in order to increase the public health benefit of prophylactic vaccination in Europe. Ultimate target is to have combined benefit among prophylactic vaccination and services in or cost effective way with maximum benefit for humanity.

## Conclusion

Increase in risk of HPV infection has a tremendous role in women related sexual problems and the hazardous effect is in the form of cervical cancer which is the major cause of death among all the cancers in developing countries. Avoidance of cervical screening and socio cultural taboos with special reference in the South East Asian belt promotes HPV and its strong relation with cervical cancer. Women are not aware, literate enough to go for regular pap screening and negative myths or facts for sexual issues in this region. Women don't go for the treatment of genital warts and the basic purpose of this review article is to highlight the dire need of awareness of this deadly cancer.

## Abbreviations

HPV: Human Papiloma virus; FTP: Full Term Pregnancy ASCUS: atypical squamous cells are of undetermined significance; CRN: cervical intraepithelial neoplasia.

## Competing interests

The authors declare that they have no competing interests.

## Authors' contributions

RF and AZ reviewed the literature, and wrote the manuscript. KK helped RF & AZ in literature review and statistical analysis. MI edited the manuscript. All the authors read and approved the final manuscript.
